# ALT control, delete: FANCM as an anti-cancer target in Alternative Lengthening of Telomeres

**DOI:** 10.1080/19491034.2019.1685246

**Published:** 2019-11-14

**Authors:** Julienne J O’Rourke, Rohan Bythell-Douglas, Elyse A Dunn, Andrew J Deans

**Affiliations:** a Genome Stability Unit, St. Vincent’s Institute of Medical Research, Fitzroy, Australia; b Department of Medicine, (St Vincent’s) University of Melbourne, Fitzroy, Australia

**Keywords:** Telomeres, DNA repair, Fanconi anemia, chemotherapy, break﻿-induced replication, precision medicine, synthetic lethal

## Abstract

Break-induced replication is a specific type of DNA repair that has a co-opted role in telomere extension by telomerase-negative cancer cells. This Alternative Lengthening of Telomeres (or ‘ALT’) is required for viability in approximately 10% of all carcinomas, but up to 50% of the soft-tissue derived sarcomas. In several recent studies, we and others demonstrate that expression and activity of FANCM, a DNA translocase protein, is essential for the viability of ALT-associated cancers. Here we provide a summary of how and why FANCM depletion leads to deletion of ALT-controlled cancers, predominantly through a hyper-activation of break-induced replication. We also discuss how FANCM can and has been targeted in cancer cell killing, including potential opportunities in ALT and other genetic backgrounds.

## Mechanism of Alternative Lengthening of Telomeres (ALT)

Telomeres consist of 5ʹ-TTAGGG-3ʹ repeats at the ends of chromosomes. They are shortened by 50–200 base pairs per cell cycle [1] because the DNA replication machinery cannot catalyze the complete replication of linear molecules (the ‘end replication problem’ [2]). Telomere shortening creates a barrier to tumorigenesis and limits the lifespan of transformed cells [3]. The majority of carcinomas and almost all hematologic malignancies subvert this barrier through activation of a reverse transcriptase enzyme called telomerase that overcomes telomere shortening by synthesizing new telomeric DNA. Telomerase is a target in cancer therapy [4,5]; however, not all cancers use telomerase to maintain telomeres. Instead, about 10% of carcinomas and at least 40% of sarcomas use a recombination/replication-based mechanism, called Alternative-lengthening of telomeres (ALT) [6]. Sarcomas that use ALT are more refractory to treatment and carry a higher risk of patient death compared to telomerase-positive tumors [7]. The ALT mechanism, which is unique to tumor cells and only rarely seen in untransformed cells [8], therefore represents a potential target for new therapies.

Cancers utilizing ALT have several unique characteristics. First, their telomeres are usually massively longer than equivalent telomerase-positive cancer cells [9]. Second, the cells contain a large amount of extrachromosomal telomeric DNA, some of which is circular and containing regions of single-stranded DNA derived from the C-rich strand (C-circles) [10]. Third, the cells display a hyper-recombinogenic phenotype in both telomeric and non-telomeric regions [11]. All of these features point to an altered DNA repair process, or at least one that is conducive to elevated telomeric recombination. In particular, the chromatin of telomeres, normally regulated by histone H3.3 and the chromatin remodeler ATRX:DAXX to be repressive to transcription and recombination, becomes highly permissive. Indeed, ATRX or DAXX (mutually exclusive) and H3.3 are obligately mutated during the development of ALT-positive cancer [11,12].

**Figure 1.**
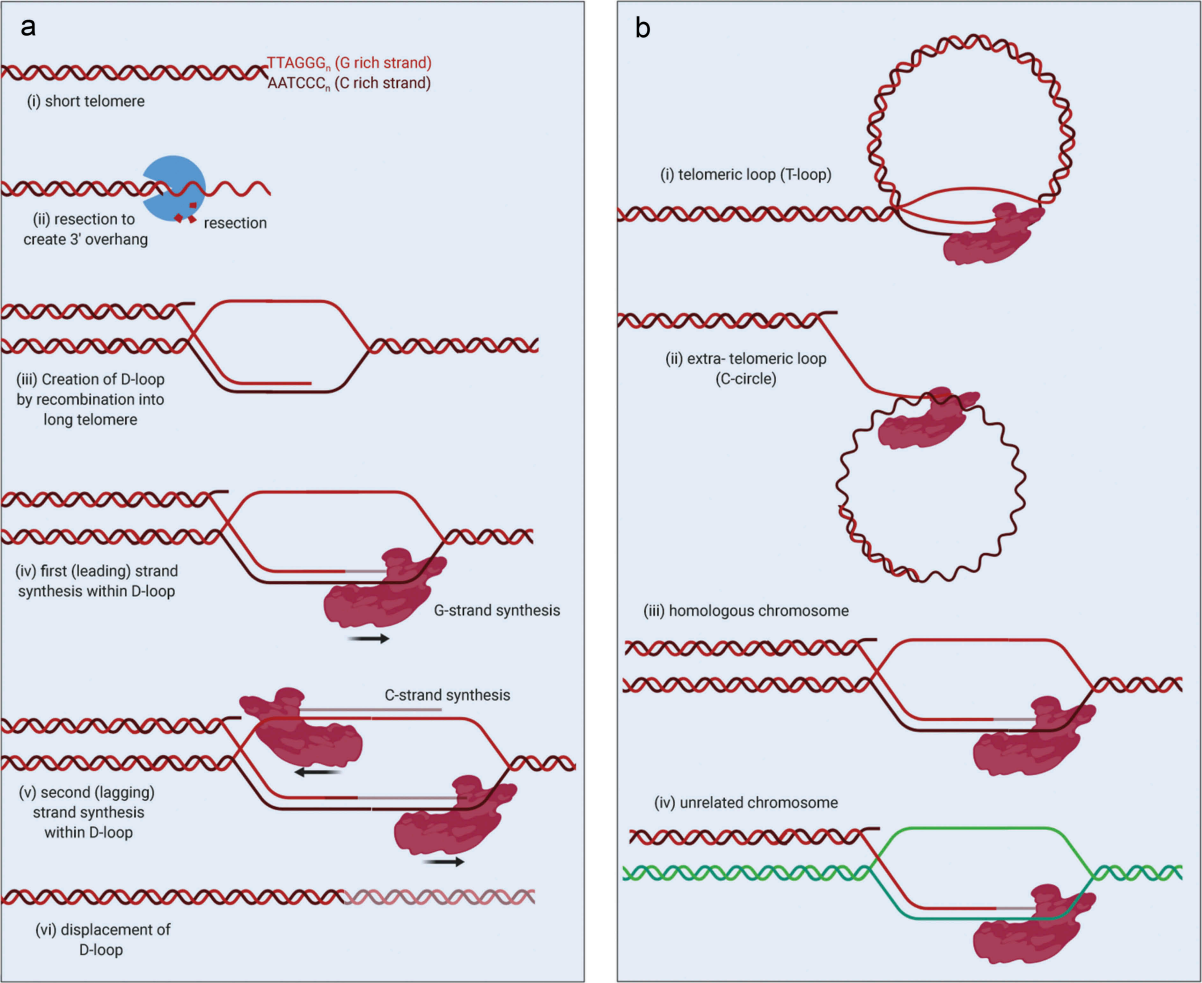
Extension of telomeres during ALT by Break-Induced Telomere Synthesis (BITS) mechanism. (a) Schematic of conservative replication of DNA by break-induced replication. (b) Four potential substrates of the proposed BITS mechanism that can lead to new telomere synthesis by ALT. Created with Biorender.com

Several recent breakthrough studies demonstrate that ‘break-induced telomere synthesis’ (BITS) (also known as telomeric MiDAS, Mitotic DNA synthesis) is necessary for the ALT mechanism [13–15]. BITS appears to require all the same factors as break-induced replication (BIR), a specialized form of homologous recombination (HR). These factors include the RAD52, BLM and POLD3 proteins [16]. In proposed models for how BITS works, the process begins with the invasion of a resected damaged telomere end (G rich-strand) into a homologous template, forming a D-loop (Figure 1(a)) [14]. In ALT, there is evidence that this template is: (i) a centromere proximal sequence of the same chromosome (T-loop), (ii) circular extrachromosomal telomeric sequences (C-circles), (iii) homologous chromosomes, or (iv) other chromosomes (Figure 1(b)). ALT may arise via usage of a combination of some or all of these templates [8,17,18]. Importantly, because telomeres are highly repetitive, invasion between or within telomeres is not limited by the requirement in HR for extended homology. After D-loop formation, DNA polymerase *δ* extends the invaded G-strand end, copying material beyond the original breakpoint, leading to initiation of lagging strand synthesis of the C-strand, also by DNA polymerase *δ* [13,19]. In canonical HR, the extension is limited by ‘second end capture’, but with broken telomere ends utilizing the aberrant templates mentioned above, there is no ‘second end’ to capture (Figure 1(a)) leading to extension of the telomere. The continued extension of the D-loop requires POLD3 and POLD4, accessory subunits of polymerase *δ* that are not essential for the normal replicative role of this enzyme. The exact role of these two components is unclear, but *in vitro* they provide increased processivity to polymerase *δ* [20]. Because BIR (and by extension, BITS) is restricted by topological constraints, increased processivity is critical for the extension of kilobases (or even megabases [9]) of telomeric DNA as a single unit.

The second feature of BITS and BIR is the production of a non-conservative DNA product; at the conclusion of the copying reaction, both strands contain entirely new DNA. This is different to canonical ‘semi-conservative’ replication, where one strand is newly synthesized, and the other comes from the original template. In this manner, BITS allows entire telomeric sequences to be copied from one chromosome to another, without affecting the length or integrity of the copied sequence. Recent work suggests that BIR proceeds via a D-loop migration model, which is supported by observation of non-conservative rather than semi-conservative products of break-induced replication at ALT telomeres [16] and the D-loop-shaped products observed by two-dimensional gel electrophoresis at sites undergoing BIR [21].

Also important to the ALT process are DNA:RNA hybrids called R-loops. R-loops form in normal telomeres at low levels but are highly elevated in ALT cells [22]. Suppression of these so-called Telomere Extended Repetitive RNAs (TERRAs) by transcription inhibition or overexpression of RNase H (which specifically degrades RNA within a DNA:RNA hybrid) leads to reduced proliferation rates in ALT cells, and shortened telomeres [22,23]. TERRA R-loops are also elevated in ATRX-/- telomerase-positive cells [24]. This indicates that R-loops could be a consequence of the relaxed chromatin environment of ALT telomeres, but many R-loop processing factors appear to play a role specifically in ALT cells [22]. There is also a possibility that TERRAs are used as a substrate to initiate break-induced replication. In bacteria, or yeast that lack telomerase, RNA-mediated replication start is a commonly used mechanism of replication akin to BIR [25,26].

Several labs have now demonstrated loss of viability in ALT cells that lack FANCM [27–29]. As FANCM is a protein that can regulate recombination through displacement of D-loops (the first step in the recombination process), replication fork stability through promotion of fork reversal, and DNA-RNA hybrid levels through displacement of R-loops, it appears to be a critical regulator of ALT.

## FANCM is a DNA repair complex anchor

**Figure 2.**
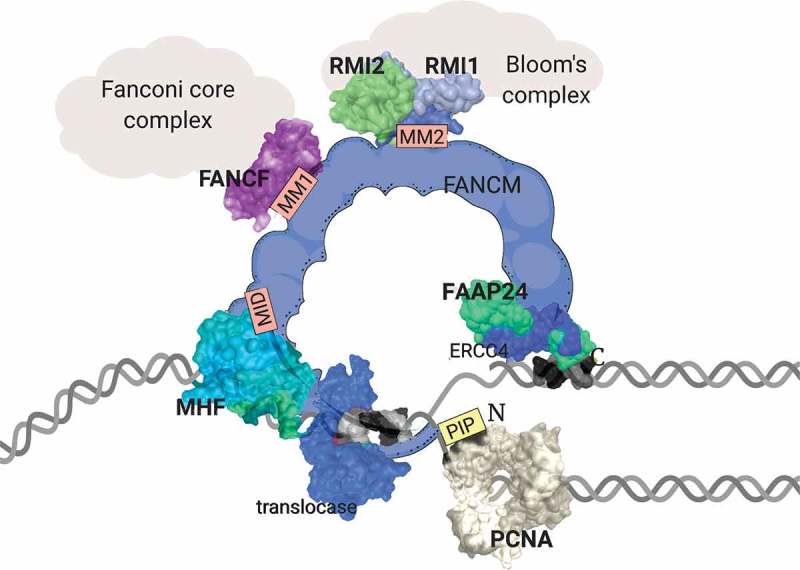
Domain architecture of FANCM protein at a replication fork. The FANCM polypeptide from N- to C-termini is shown in blue. Key domains are highlighted, including N-terminal PIP box that binds PCNA, MHF-interaction domain (MID), FANCF interaction domain (MM1) and RMI1–RMI2 interaction domain (MM2). The DNA binding translocase and ERCC4 domains (bound to FAAP24) are shown in dark blue. Where available, crystal structures are shown. Scale and arrangement are only an approximation. Created with Biorender.com.

FANCM is a large, 2048 amino acid protein with multiple DNA binding domains and protein:protein interaction motifs (Figure 2). In particular, FANCM has been shown to bind DNA in a structure-specific manner [30]. Two DNA binding domains exist in the protein: an N-terminal DEAH domain required for recognition of fork-shaped DNA (described further below), and a C-terminal ERCC4 pseudo-nuclease domain, required for localizing the protein to specific DNA damage sites. Despite evolutionary similarity to restriction endonuclease domains, the ERCC4 domain does not cleave DNA, but instead binds structures containing dsDNA:ssDNA junctions [31]. EM investigations suggest that the N- and C-terminal DNA binding domains come together in the overall architecture of the protein [32]. Further DNA binding activity of FANCM comes from the association with FAAP24 (another ERCC4 domain-containing protein), and MHF1 and MHF2 (histone-fold containing proteins that promote the association of FANCM with DNA junctions) [33]. The extensive unstructured sequence between the N- and C-terminal DNA binding domains contains several protein:protein interactions sites, indicating a scaffold role in the recruitment of multiple protein complexes. In particular, this includes binding sites for complexes involved in the cancer predisposition disorders Fanconi anemia (the Fanconi core complex) and Bloom’s syndrome (the Bloom’s complex) [34] (Figure 2).

FANCM is so-named as it was first identified as homozygous mutated in an individual with Fanconi Anemia [35]; however, it was later demonstrated that this FA patient also had homozygous FANCA deficiency [36]. None-the-less, multiple lines of evidence demonstrate the FANCM does participate in the ‘FA-BRCA pathway’, in which the activity of at least 22 FANC proteins, including BRCA1 and BRCA2, converge on the conjugation of a single ubiquitin to the chromatin-associated FANCD2 protein [37]. Monoubiquitinated FANCD2 forms ‘foci’, the discrete localization of many molecules of FANCD2 at damaged replication forks, that signals further repair processes. FANCM is necessary for the complete activation of FANCD2 monoubiquitination, and essential for FANCD2 foci formation in human, mouse and chicken cells [34,38,39]. In addition to its role in the FA-BRCA pathway, FANCM also regulates and activates the Bloom’s complex.

The Bloom’s complex consists of four proteins: BLM (a helicase), TOP3A (a type 1A topoisomerase) and RMI1 and RMI2 (oligonucleotide-binding fold proteins). Mutation in any one of these proteins leads to the cancer predisposition disorder Bloom’s Syndrome [40–42]. The tumor-suppressor function of Bloom’s complex is likely due to its important role in genetic recombination. The Bloom’s complex suppresses recombination by promoting Holliday junction ‘dissolution’, a process that unlinks catenated DNA recombination intermediates formed during homologous repair, for example, back to their pre-recombination state [43]. BLM also suppresses recombination by promoting break-induced replication, which may be mediated by interaction with POLD4 (aka p12) and stimulation of polymerase *δ* activity on D-loops [44]. Bloom’s complex activity is also critical for the ALT mechanism [45]. For example, in the absence of BLM, telomeres are cleaved by the SMX (SLX4/Mus81/XPF) Holliday junction nuclease complex leading to senescence [18].

FANCM physically connects the FA core complex and the Bloom’s Complex to form a nuclear complex termed BRAFT, indicating that the BLM and FA pathways are connected in genome maintenance [46]. In previous studies, the binding of the FA core complex and the Bloom’s complex was shown to be mediated by two different fragments of FANCM [34]. The first fragment corresponding to residues 687–1104 contains the MM1 domain, which binds and recruits the FA core complex to DNA damage sites. A second FANCM fragment, corresponding to residues 1027 to 1362 interacted specifically with the Bloom’s complex components RMI1 and RMI2 [34,47]. Within this fragment, a conserved MM2 domain was identified. Removal of the MM2 domain from full-length FANCM (FANCMΔ1209-1251) resulted in complete loss of interactions with the Bloom’s complex [48]. Within the MM2 motif, four phenylalanine residues and a string of acidic residues are highly conserved [34,47]. The X-ray crystal structure of both RMI1 and RMI2 bound to the MM2 peptide shows that key phenylalanine and other hydrophobic residues of the MM2 domain interact with RMI1 by a ‘knobs into holes’ arrangement. Mutation of each of these hydrophobic MM2 residues to alanine significantly decrease interaction of FANCM with the Bloom’s complex, demonstrating the importance of this binding motif [47].

## FANCM is a motor protein that promotes DNA branch migration

FANCM contains an ATPase motor domain at the N-terminus of the protein (residues 64–684), where the two RecA-like folds are separated by an insertion domain (residues 298–433). FANCM falls within the RIG-I-like family of SF2 helicases, related to yeast Mph1 and archaeal HEF helicases [49]. These ATPases preferentially bind to and unwind branched oligonucleotide molecules [50]. For Mph1 and HEF, direct ATP-dependent unwinding of junction DNA to ssDNA (i.e. *bona fide* helicase activity) has been observed [50,51]. However, true helicase activity has not been observed for FANCM, which instead uses its ATPase motor to perform branch migration by translocation [30]. Translocation and helicase activity are similar processes as both depend on the concerted ATPase and DNA binding activity of the two RecA-like domains, which move the protein along the DNA molecule via an ‘inchworm’ mechanism [52]. The distinction between the two is the presence of a ‘wedge’ domain in helicases [53], which separates the two strands of DNA while the protein translocates, effectively peeling the two strands apart with each base pair step of translocation. FANCM on the other hand, facilitates branch migration by pushing the branchpoint of the junction as the protein translocates along DNA, without the simultaneous generation of ssDNA. FANCM, therefore, lacks a ‘snow plow’ domain but possesses a DNA binding domain specific for branched structures. The coupling of this structure-specific DNA binding domain together with the translocase activity of the ATPase motor domain is what permits FANCM to migrate branched DNA structures.

**Figure 3.**
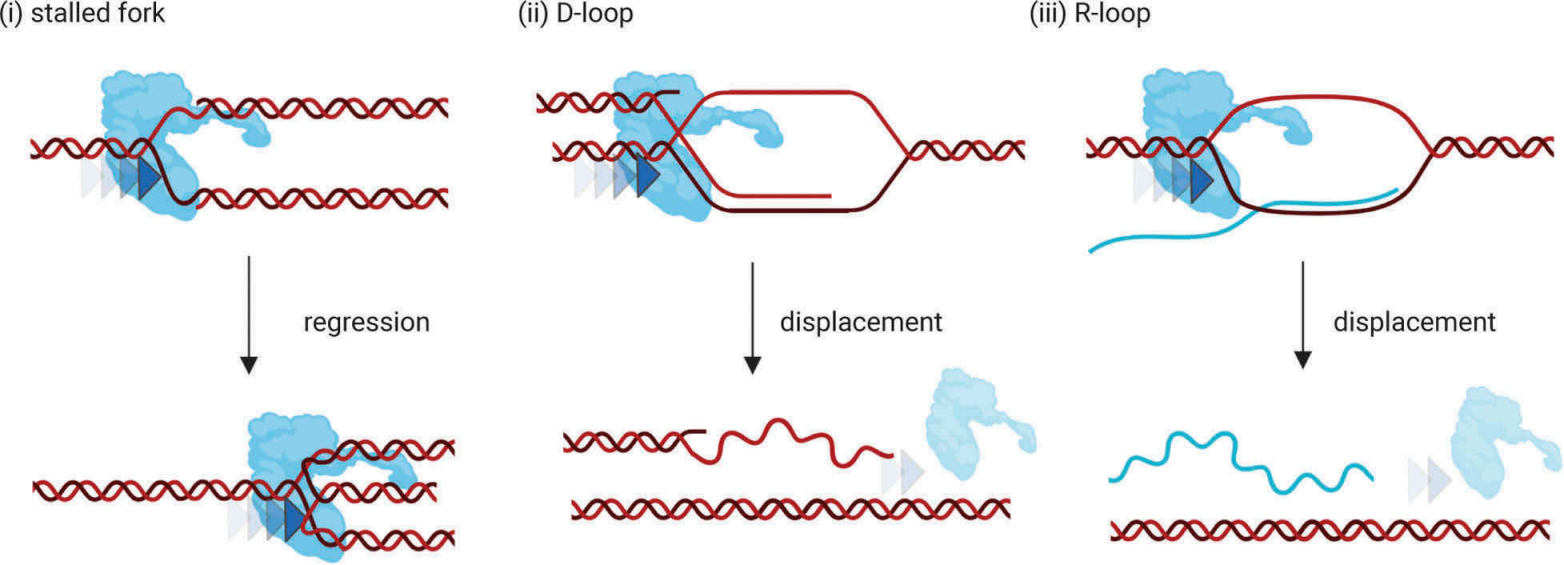
Branch migration of different DNA junction structures by FANCM and their associated products. Direction of branch migration by FANCM (blue protein) is indicated by blue arrows. Created with Biorender.com.

Dependent upon the nature of the junction that is migrated by FANCM, several different outcomes are possible (Figure 3). First, the branch migration of stalled replication forks by FANCM can lead to replication fork reversal (Figure 3(i)). Fork reversal leads to formation of a more stable four-way junction (also known as a chicken-foot structure, which is structurally identical to a Holliday junction) that protects the DNA and permits replication restart without nuclease cleavage after repair is completed [54]. Second, the branch migration of recombination-mediated displacement loops (D-loops) by FANCM can suppress recombination [30] (Figure 3(ii)). Third, branch migration of three-stranded DNA:RNA hybrid structures by FANCM can suppress the persistence of R-loops [55] (Figure 3(iii)). This could have effects on transcription, recombination, or the removal of R-loops as barriers to DNA replication. There is *in vitro* and cell-based evidence that FANCM deficiency allows accumulation of unregressed replication forks [56], recombination intermediates [57] and R-loops [55].

## Proposed role of FANCM in BIR, BITS, and ALT

As outlined above there are many possible mechanisms by which FANCM could limit cellular toxicity of the ALT phenotype. However, the data from our published studies suggest that the dominant function is mediated by FANCM interaction with Bloom’s complex, to suppress break-induced replication (BIR) at telomeres [28,29]. Our results are reminiscent of those observed in yeast, where Sgs1 (BLM-homolog) and Mph1 (FANCM homolog) positively and negatively regulate break-induced replication, respectively [58]. Mph1 and Sgs1 co-purify from yeast [59], however in vertebrates the two enzymes have acquired a further integration of function through acquisition of RMI2, a protein that bridges the two complexes [47], and the MM2-domain in FANCM [34]. This integration of BLM and FANCM in a single complex supports the idea of a combined role in break-induced replication. Importantly, BLM also directly binds to the POLD4 subunit of polymerase δ to promote the *in vitro* processivity of this enzyme on D-loops [44].

**Figure 4.**
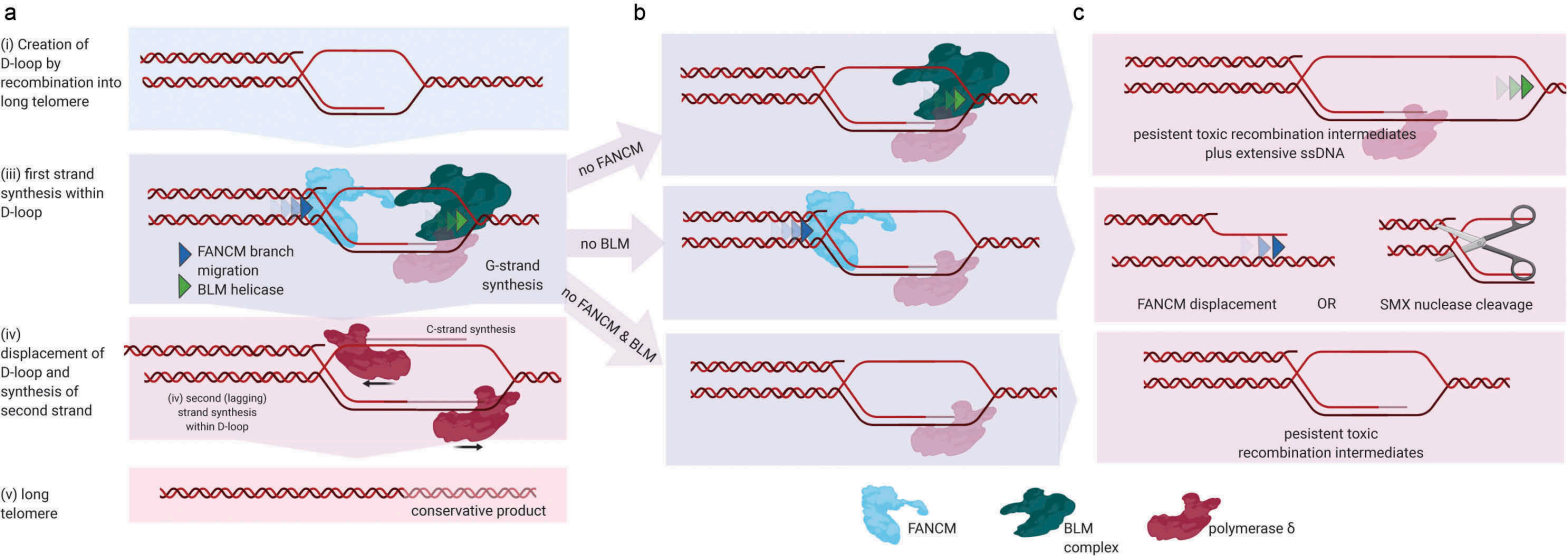
Proposed model for bubble branch migration and telomere synthesis by BLM, FANCM and pol δ. (a) Steps (i)-(ii) FANCM and Bloom’s complex are recruited as a complex to the D-loop. BLM helicase promotes DNA unwinding in direction of green arrows, to promote DNA synthesis of G-strand by polymerase delta. FANCM branch migration promotes dissociation of nascent DNA in direction of blue arrows. Steps (iii)-(iv) C-strand synthesis completes the generation of new telomeres. (b) step (ii) but in absence of either BLM, FANCM or both FANCM and BLM. (c) Outcomes of aberrant pathway. Created with Biorender.com.

Why would FANCM and BLM be brought together to function within the same complex? We put forward a hypothetical model (Figure 4). In this model, FANCM and BLM work cooperatively in linking anti- and pro-recombination functions of each enzyme into a machine that promotes D-loop migration, and DNA synthesis by polymerase δ during BIR (Figure 4). First, Bloom’s complex branch unwinds and *extends* D-loop structures. In doing so, BLM may act in place of the replicative MCM helicase, similar to what has previously been proposed for Pif1 helicase [60]. Second, BLM unwinding stimulates polymerase δ to synthesize new G-strand leading strand DNA toward the end of the chromosome, using the C-rich strand of the telomere as a template. Third, pol ⍺ primase and additional polymerase δ synthesize nascent lagging C-strand DNA, followed by the displacement of the now conservatively duplicated DNA. This displacement could also be BLM and/or FANCM mediated (Figure 4). Finally, FANCM acts to couple these leading and lagging strand synthesis steps. It does so by reversing or ultimately displacing any stalled ‘BIR replisome’. This is analogous to the canonical functions of FANCM in replication fork reversal and D-loop dissociation [30,54], except that it is now tethered directly to a BIR replisome together with polymerase δ and BLM complex in a migrating D-loop. In the context of an ALT-telomere, such stalling would most likely be due to additional damage on the C-rich leading strand template, which experimental evidence indicates is far more labile than the G-rich lagging strand template [61]. We propose that FANCM-mediated D-loop dissociation in the context of a BIR replisome would prevent uncontrolled lagging strand synthesis of the C-strand. This model requires no new activities, just the physical coupling of known functions of both BLM and FANCM.

The model proposed is wholly supported by the outcomes observed when one or other or both of FANCM and BLM are absent in ALT cells. In particular, we observed a large accumulation of C-strand ssDNA in FANCM deficiency, which is not due to new telomere synthesis [28]. This C-rich ssDNA must therefore originate from abnormally long regions of polymerase δ displaced template C-strand, and not newly synthesized C-strand. This C-rich DNA, based on the model above, is hypothesized to originate from uncoupled lagging strand synthesis upon leading strand stalling. Indeed, our data using two-dimensional electrophoresis revealed a novel C-strand telomeric structure that uniquely appears upon FANCM depletion, and conceivably represents such an ssDNA region contained within a large D-loop structure. It is also possible that FANCM could directly displace RAD51-dependent or independent D-loop structures to prevent them being engaged by BIR at all. However, counter to this hypothesis, FANCM retains the ability to directly unwind D-loops even without an intact Bloom’s complex interaction domain [62]. Experimental models using recombinant proteins or cells with defined genetic mutations could be used in the future to further demonstrate the feasibility of our model.

Other phenotypes of FANCM deficiency could also be explained by a role for the protein in D-loop displacement coupled with BIR. First, recent studies have shown that FANCM can preserve the integrity of common fragile sites [63] and centromeric repeats [64] through an FA pathway independent function. Neither of these studies examined whether decreased stability of such repeats in the absence of FANCM was dependent upon BLM, but another study has shown that FANCM/BLM co-depletion can greatly amplify tandem duplication events during a BIR dependent process called replication bypass restart [65]. Second, FANCM recruitment to forks actually requires interaction with the Bloom’s complex, something which has also been demonstrated in telomerase-positive cells [66]. Third, the elevated meiotic crossovers (particularly nearer chromosome ends) observed in FANCM knockout organisms [67,68] could be due to an anti-BIR rather than a pro-HR role for FANCM in meiosis; although this requires further investigation.

## FANCM as a therapeutic target in cancer treatment?

There are several potential ways in which FANCM activity could be targeted as an anti-cancer agent. In the context of ALT, we have demonstrated that one of the best targets may be the MM2 domain. Inhibiting the interaction of FANCM and the Bloom’s complex with ectopic MM2 peptide (that acts as a dominant decoy) was sufficient to inhibit colony formation of ALT-associated cancer cells, but not telomerase-positive cancer cells [28]. This peptide works as a dominant interfering binder to RMI1:RMI2, and sequesters the Bloom’s complex away from FANCM [34]. As with FANCM depletion, this induces death through a ‘hyper-ALT’ phenotype, leading to increased telomeric ssDNA, C-circles and TIFs [28]. A recent *in vitro* high-throughput screen for small molecule inhibitors of MM2–RMI1:2 interaction lead to the discovery of PIP-199 [69]. We found that this experimental drug also showed some discriminatory activity in killing ALT-cells, compared to telomerase-positive cells [28]. The compound does appear to be more toxic overall than the peptide, probably owing to the fact that it has not been further optimized for cell-based use. Further development of PIP-199, or the identification of other MM2 mimetics would be a valuable strategy for ALT-based therapy.

Several other domains and functions of FANCM may also be amenable to drug development. Existing crystal structures of FANCM domains bound to FAAP24 [32] or MHF1:MHF2 heterotetramer [70] could be screened *in silico* against protein:protein interaction inhibitors. Alternatively, the ATPase motor domain activity is also essential for suppression of ALT, and could be screened against chemicals that target this enzyme fold [71]. Other approaches such as epitope-targeted degradation [72], siRNA based knockdown [73], or other target-tailored approaches to reduce FANCM expression levels are also potential strategies.

In addition to ALT, several other synthetic lethal interactions have been observed for FANCM that may widen the targetability of the protein in therapeutic use. The evidence and implications of some of these are highlighted in Table 1.

**Table 1.** Other genetic interactions with FANCM deficiency.

Gene	Genetic interaction	Evidence and implications	Citation
*BRCA1*	Synthetic lethal	FANCM and BRCA1 co-siRNA promotes increase in tandem duplications and cell death.	[27,65]
*RAD52*	Synthetic lethal	RAD52 shRNA in FANCM-/- HCT116 cells leads to loss of colony formation and loss of tumor formation in nude mice.	[63]
*XPF*	Synthetic lethal	XPF shRNA in FANCM-/- HCT116 cells leads to loss of colony formation. Elevated recombination intermediates in FANCM-/- cells is cleaved by XPF.	[74]
*WEE1*	Synthetic lethal	FANCM siRNA and WEE1 inhibitor combined in various cancer cell lines. WEE1 inhibition triggers direct mitotic entry without completing DNA synthesis, resulting in catastrophic chromosome fragmentation and apoptosis	[75]
*BLM*	Synthetic lethal	BLM shRNA in FANCM-/- HCT116 cells leads to loss of colony formation. Additive lethality in BLM siRNA treated ALT cells has also been reported.	[27,76]
*FANCD2, FANCC*, other FANC genes	Synthetic rescue	FANCM siRNA identified in a screen for rescuers of FANCD2- or FANC-deficient HAP1 cells. Suggests FANCM creates toxic intermediate during ICL repair that must be resolved by rest of FA pathway.	[77]
*PARP1*	Sensitivity	FANCM deficiency was shown to be a determinant of sensitivity to PARP inhibition	[78]

Importantly, FANCM deficiency can be tolerated in telomerase-positive human cancer cell lines such as HEK293 [34], HeLa [79],and HCT116 [80]. Absence of FANCM is also compatible with normal development in model organisms such as mouse [39], fruit fly [81], and arabidopsis [67]. Presumably, FANCM also restricts BIR or BITS in telomerase-positive cells, so why is its deletion specifically lethal in ALT? Some data suggest that heterochromatin acts as an additional barrier to ‘runaway’ DNA synthesis by BIR [82,83]. The altered chromatin state of ALT-positive cells means that this barrier is no longer intact, permitting more extensive BIR activity in the absence of FANCM. The genetic and biochemical evidence for a role of FANCM in unleashing a toxic ‘hyper-ALT’ phenotype of cancers using alternative lengthening of telomeres suggest that any experimental approach that disrupts the BLM-FANCM complex is likely to have therapeutic potential. The vital role of FANCM in ALT make it an excellent target candidate in these difficult to treat cancers.
